# Implantable collamer lens surgery for high myopia: safety boundaries, vault management, and patient selection

**DOI:** 10.3389/fopht.2026.1908210

**Published:** 2026-10-27

**Authors:** Zitong Yu, Tong Qin

**Affiliations:** 1 Shanghai Bright Eye Hospital, Shanghai, China; 2 Shanghai Jiao Tong University, Shanghai, China

**Keywords:** artificial intelligence, EVO ICL, hyperopia, implantable collamer lens, IPCL, lens power calculation, phakic intraocular lens, vault

## Abstract

Posterior chamber implantable collamer lens (ICL) implantation provides additive refractive correction for selected phakic patients, especially those with high myopia and limited corneal laser options. This narrative Mini Review synthesizes literature published mainly from January 2021 through May 2026 to connect patient selection with measurable safety boundaries, separate lens power and size planning, dynamic vault assessment, and long-term surveillance. It also discusses hyperopic ICLs, non-EVO lenses, alternative phakic IOL platforms such as IPCL, and emerging artificial intelligence. True anterior chamber depth, age-appropriate endothelial reserve, open angles, crystalline lens status, and refractive stability define eligibility. Central vault is interpreted using pragmatic numerical categories alongside angle, pressure, and lens findings; no single vault threshold guarantees safety or mandates exchange. Refractive and vault prediction models have different inputs and outcomes, and favorable average accuracy does not establish performance in extreme or underrepresented anatomy. Modern central-port ICLs show good outcomes in appropriately selected myopic eyes, while evidence for hyperopic and alternative platforms is smaller and not interchangeable. The proposed clinical framework represents expert synthesis rather than a validated decision rule. Future priorities include externally validated multimodal prediction and prospective lifelong safety monitoring.

## 1 Introduction

Posterior chamber implantable collamer lens (ICL) surgery provides additive refractive correction while preserving the crystalline lens and corneal tissue. Central-port EVO/EVO+ designs have broadened its role in moderate to high myopia, particularly when corneal thickness, topography, ocular surface status, or optical demands limit laser surgery (1–5). However, removability does not reverse every complication: cataract, endothelial loss, glaucoma, and additional intraocular surgery remain relevant risks.

This review links candidate selection to measurable safety boundaries, lens power and size selection, dynamic vault assessment, and lifelong surveillance. High myopia remains the principal focus, with hyperopic ICLs and alternative phakic intraocular lenses (pIOLs) included to clarify the limits of extrapolating EVO evidence. The proposed clinical framework is an expert narrative synthesis, not a prospectively validated decision tool.

## 2 Search strategy and scope

The narrative literature review prioritizes English-language articles published from January 2021 through May 2026, with no lower date restriction for foundational device studies and long-term reports. This is the publication eligibility window, rather than a period of patient data collection. PubMed/MEDLINE was the principal source, supplemented by reference lists and publisher pages, including Frontiers. Search terms included implantable collamer lens, EVO/EVO+, V4c/V5, toric ICL, vault, sizing, endothelial cell density, cataract, intraocular pressure, high myopia, SMILE, and LASIK. During revision, targeted searches added hyperopia, IPCL, other phakic IOLs, power calculation, predictive formula, artificial intelligence, and machine learning; the May 2026 publication cutoff was retained.

Clinical cohorts, comparative studies, prediction-model studies, systematic reviews, and meta-analyses were considered when they addressed selection, visual/refraction outcomes, sizing, vault, or complications. Case reports informed unusual safety mechanisms, not incidence estimates; regulatory documents clarified device-specific eligibility. Duplicate publications, nonclinical material without relevant design information, and reports outside scope were not retained. Evidence was synthesized by clinical domain, considering device generation, study design, follow-up, sample size, and validation setting. **Figure 1** describes this qualitative selection pathway. No formal PRISMA screening, reconstructed screening counts, quantitative pooling, or formal risk-of-bias assessment was undertaken.

**Figure 1:**
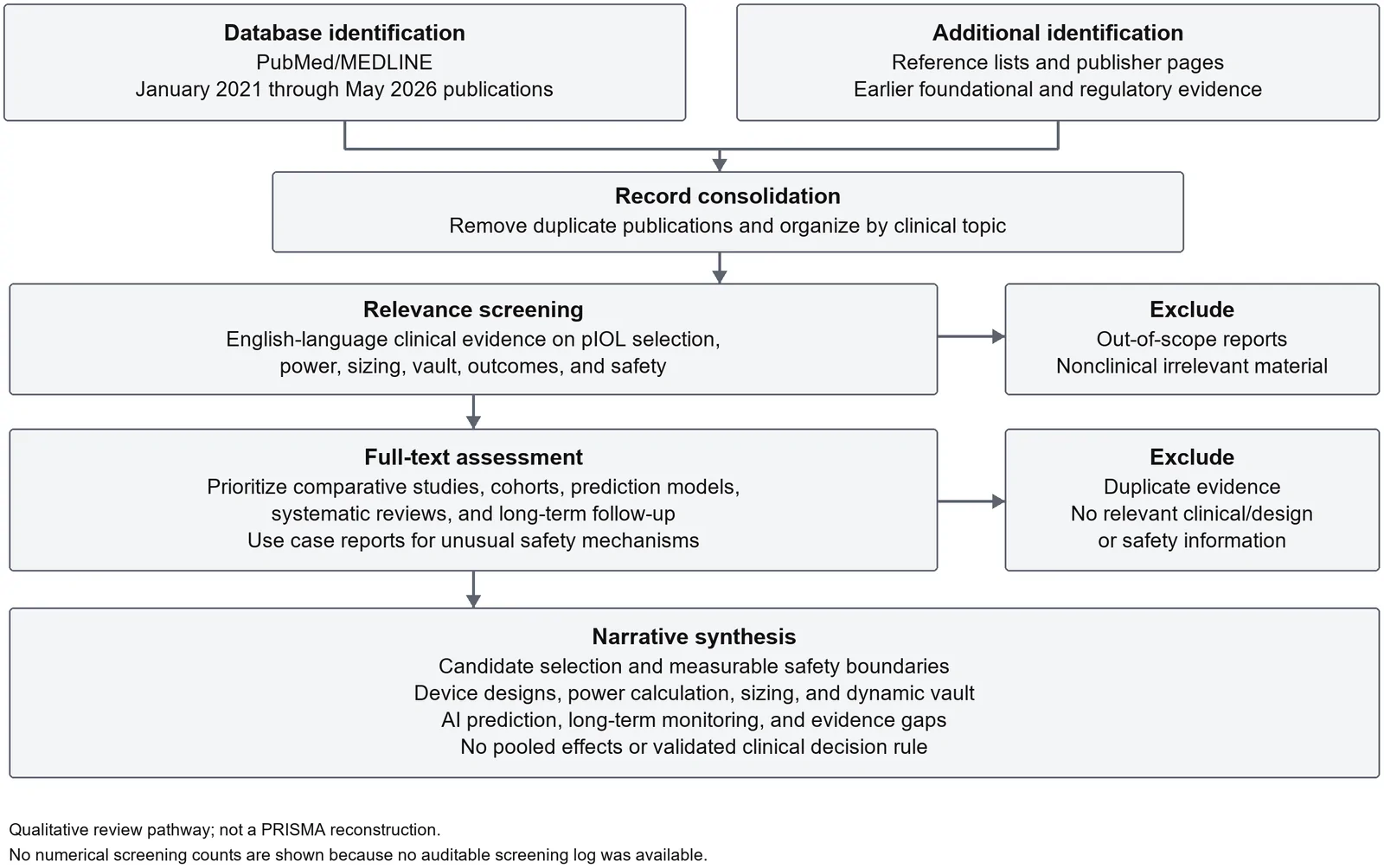
Literature search and selection. Qualitative identification, relevance assessment, full-text selection, and thematic synthesis for this narrative review. The publication window was January 2021 through May 2026, with earlier foundational and regulatory evidence considered where relevant. No auditable screening log was available; numerical PRISMA-style counts were therefore not reconstructed. The flowchart describes the review pathway and does not imply systematic-review completeness.

## 3 Why ICL is a distinct refractive category

SMILE, femtosecond laser-assisted LASIK (FS-LASIK), and transepithelial photorefractive keratectomy (TransPRK) alter corneal shape; ICL adds a posterior chamber optical element. Tissue preservation can be valuable in high corrections, but must be balanced against intraocular infection, inflammation, cataract, endothelial, and pressure-related risks (6–8). The 0.36-mm central port of EVO/EVO+ permits aqueous flow and removes the routine need for prophylactic peripheral iridotomy for these models; it does not eliminate pupillary block or sizing-related complications (3, 4, 9).

## 4 Patient selection and preoperative safety

Suitable candidates have stable refraction, a clear crystalline lens, adequate true anterior chamber depth (ACD), open angles, and age-appropriate endothelial reserve. **Table 1** summarizes preoperative boundaries and postoperative monitoring. True ACD is measured from corneal endothelium to the anterior lens surface; an epithelial-reference measurement must not be used interchangeably. Thresholds depend on the model and jurisdiction. For US EVO labeling, true ACD must be at least 3.0 mm; minimum endothelial cell density (ECD) varies with both age and ACD (9).

**Table 1:** Preoperative and postoperative safety boundaries.

Domain	Preoperative boundary	Postoperative assessment
Age and stability	Follow local labeling. US EVO age range: 21–60 years; stable refraction within 0.50 D for at least 1 year (9, 10).	Review age-related lens change and refractive drift; counsel on presbyopia and future cataract surgery.
True ACD and angle	US EVO: ACD >=3.0 mm from endothelium to lens; gonioscopic angle at least grade III. Other device/jurisdiction thresholds differ (9, 18).	Assess angle crowding and peripheral clearance with gonioscopy/AS-OCT; a central vault alone does not establish safety.
Endothelial reserve	Use the model-specific age/ACD matrix. Example US EVO, age 26-30: minimum ECD 3,425, 3,375, and 2,900 cells/mm2 for true ACD >=3.0, >=3.2, and >=3.5 mm, respectively (9).	Serial specular microscopy using comparable methods; investigate sustained decline, abnormal morphology, or edema (18, 36).
Crystalline lens	Clear lens; assess lens rise, configuration, and anticipated cataract risk (6, 8).	Monitor opacity and lens contact; evaluate symptoms and progression before deciding on exchange/explantation.
IOP and glaucoma	No uncontrolled glaucoma, narrow-angle disease, or unexplained ocular hypertension; normal IOP does not exclude glaucoma (9).	Check IOP at 1–6 hours per US directions; reassess early and during steroid treatment. New pressure rise, pain, or angle closure warrants prompt evaluation (9, 37).
Central vault	Compare multimodal measurements and plausible sizes; do not rely on a single nomogram (20–22).	Low <250; common target 250-750; high >750-1,000; excessive >1,000 micrometers. <100 or lens contact is particularly concerning. Interpret with lens clarity, angle, and IOP (22, 27, 28).
Retina and follow-up	Document retinal/macular status, visual potential, and capacity for continued surveillance (5, 31).	Continue review of vault, ECD, IOP/angle, lens clarity, refraction, and symptoms. New flashes/floaters or field loss needs urgent retinal assessment.

Regulatory examples refer to the US EVO platform; clinicians must use the current labeling for the actual model and jurisdiction. ECD examples are minimum eligibility values, not targets. Vault categories are pragmatic reporting ranges, not automatic intervention rules. Long-term visit frequency should be individualized to anatomy, symptoms, and observed trends.

US approval expanded the EVO/EVO+ ICL/TICL age range from 21–45 to 21–60 years in November 2025 (10). This regulatory range does not establish suitability for every older patient. Lens opacity, lens rise, angle crowding, presbyopia, and the likely interval to cataract surgery require separate assessment. A fixed age threshold around 40 years should therefore not determine the choice between ICL and lens extraction. Patients must also understand persistent high-myopia retinal risk and commit to long-term follow-up (5, 8, 11, 12).

### 4.1 Hyperopic ICLs

Hyperopic ICL can be considered for selected accommodating adults with stable moderate to high hyperopia when corneal correction is unsuitable and anterior segment anatomy meets the relevant lens labeling. Cycloplegic refraction helps identify latent hyperopia. Available published designs include spherical ICH V3 and toric V4B lenses; availability and approval vary by country. These positive-power, non-EVO lenses should not inherit the central-port claims or myopic sizing assumptions of EVO. Peripheral iridotomy is required for the non-port designs (13–15).

Hyperopic eyes often have shorter axial length, shallower chambers, and narrower angles; positive-power optics and crystalline lens configuration can further constrain space. Vault must therefore be interpreted alongside peripheral lens clearance and angle anatomy. There is no robust evidence that hyperopic vault is uniformly higher or lower than myopic vault: a small older-model cohort reported similar long-term central vault in the two groups (13). Amblyopia, latent hyperopia, and limited toric availability can also constrain visual gains and predictability.

In 20 eyes receiving hyperopic toric ICL, mean vault decreased from approximately 650 micrometers at one month to 613 micrometers at one year (14). A 2025 single-arm study of spherical ICL followed by LASIK in 48 hyperopic astigmatic eyes reported 93.8% within +/-0.75 D of target, but assessed the combined procedure over short follow-up (15). These findings cannot establish equivalence to myopic EVO outcomes or long-term safety. A shallow or narrow-angle hyperopic eye remains unsuitable for pIOL implantation despite a large refractive error.

## 5 Non-EVO ICL and alternative phakic IOLs

Earlier non-port Collamer ICLs use posterior chamber plate-haptic support and require an alternative aqueous pathway through peripheral iridotomy. IPCL V2.0 instead uses a hydrophilic acrylic platform with a different haptic geometry and a central port in the studied myopic design; spherical, toric, hyperopic, and diffractive options are model- and market-dependent (2, 16–18). Both posterior chamber platforms vault through the interaction of lens geometry and anatomical support. Identical lens lengths, powers, or vault nomograms cannot be assumed interchangeable.

A randomized comparative study of 60 myopic eyes found visual improvement with both central-port ICL and IPCL over 12 months, without a detected difference in reported adverse effects; its size and duration cannot exclude differences in rare or late complications (17). Eyecryl is another hydrophilic acrylic posterior chamber platform, whereas Artisan/Artiflex lenses use anterior chamber iris fixation. Their endothelial clearance and fixation-related risks differ from posterior chamber vault concerns (18, 19). **Supplementary Table S1** compares design, optics, indications, support, and evidence limitations. Available comparisons support individualized selection, not device equivalence.

## 6 Preoperative imaging and lens sizing

Traditional sizing uses white-to-white distance (WTW) and ACD as surrogates for posterior chamber anatomy. Anterior segment optical coherence tomography (AS-OCT) and ultrasound biomicroscopy (UBM) add anterior chamber width (ACW), angle-to-angle distance (ATA), sulcus-to-sulcus distance (STS), crystalline lens rise (CLR), and iridociliary morphology (1, 20, 21). Measurements from different devices may yield different size recommendations (20).

The NK formula incorporates ACW and CLR with lens size; the KS approach uses ATA and lens size. In a 106-eye comparison, achieved-minus-predicted vault bias was -104.82 micrometers for NK and -68.55 micrometers for KS, with wide agreement limits and no significant overall difference in absolute prediction error (22). Thus a smaller mean bias does not establish greater precision. Haptic placement, iris shape, and inter-eye differences warrant multimodal review, especially when two adjacent sizes appear plausible (23–25).

## 7 ICL power calculation and refractive prediction

Power selection determines refractive correction; sizing and vault prediction determine anatomical fit. These tasks require distinct endpoints. Manufacturer power calculations use a modified vergence approach based on spectacle-plane refraction, vertex distance, keratometry, ACD, and target refraction; toric planning additionally considers cylinder and axis (1, 26). Repeatable manifest and cycloplegic measurements and correct vertex conversion are especially important at large refractive errors. Cataract IOL formulas should not be substituted directly because the natural lens remains *in situ*.

Jiang et al. evaluated 2,767 eyes using stacked regression models with refraction, age, ocular biometry, and implanted lens parameters. The best spherical-equivalent prediction mean absolute errors were 0.339 D for non-toric ICL and 0.325 D for toric ICL; overall accuracy was comparable to the modified vergence formula, with possible advantages concentrated in low-to-moderate myopia (26). This does not demonstrate superiority in extreme myopia, hyperopia, or atypical chambers. **Supplementary Table S2** separates refractive and vault models, their inputs, and reported accuracy. No universally superior formula is established for these uncommon phenotypes.

## 8 Vault as a dynamic safety biomarker

Central vault is the perpendicular separation between the posterior ICL surface and anterior crystalline lens surface. For consistent terminology, this review defines low vault as <250 micrometers, a commonly targeted range as 250–750 micrometers, high vault as >750 to 1,000 micrometers, and excessive vault as >1,000 micrometers; <100 micrometers or lens contact deserves particular concern (22, 27, 28). These pragmatic categories are not validated universal intervention thresholds, and published acceptable ranges differ (1).

Low vault raises concern for lens contact and anterior subcapsular opacity; excessive vault may crowd the angle and elevate intraocular pressure (IOP). A low stable vault without contact or opacity, or a high vault with open angles and normal IOP, may be observed. Lens touch, progressive cataract, angle closure, pigment dispersion, or pressure elevation requires mechanism-based reassessment, potentially including repositioning, exchange, or explantation (6, 8, 29). Management must not be triggered by a central number alone.

Illumination, pupil diameter, accommodation, imaging device, and postoperative timing affect vault. Serial measurements should use comparable conditions and include angle assessment. Posterior chamber morphology, haptic position, lens aging, and rotation explain changes over time and differences between fellow eyes (19, 23, 25, 30).

## 9 Visual and refractive outcomes

Reviews and cohorts support good efficacy and predictability in selected high and super-high myopic eyes (7, 31). Comparisons with SMILE suggest possible optical advantages for ICL at higher corrections, but selection bias, baseline differences, and heterogeneous endpoints limit procedure rankings (32, 33). Corneal procedures avoid intraocular risks and may suit lower corrections with favorable corneal parameters. Glare, halos, mesopic pupil size, and subjective visual quality remain relevant even with excellent acuity.

Toric ICL offers correction of regular astigmatism, but residual cylinder depends on measurement repeatability, axis alignment, and postoperative rotation (34). Vertical placement has been investigated to improve stability; it is not a universal recommendation and must respect model-specific axis planning (35).

## 10 Safety of the endothelium crystalline lens and IOP

Safety assessment includes ECD, lens transparency, IOP, angle configuration, inflammation, infection, retinal status, and reoperation. Meta-analyses support favorable outcomes in selected eyes while demonstrating nonzero complication risks (7, 8). Endothelial loss may be clinically silent; baseline reserve, measurement reliability, surgical trauma, and longitudinal trends matter more than a single acceptable postoperative count (36). Cataract remains relevant despite central-port design, particularly with older age, lens contact, or adverse lens anatomy (4, 27).

Early pressure elevation can reflect retained viscoelastic, pupillary block, or excessive vault, whereas later elevation may reflect steroid response or pigment-related mechanisms. FDA directions advise an initial IOP check within 1–6 hours after implantation; subsequent surveillance should follow clinical findings and local protocols (9, 37). Adverse-event databases identify safety signals but cannot estimate incidence or compare devices reliably without denominators and control for reporting bias (38, 39).

Reports of late glaucoma with first-generation pIOLs and perioperative drug-related maculopathy illustrate surveillance and operative hazards; they should not be used to quantify current EVO risk (40, 41). Patients developing cataract may need ICL removal with phacoemulsification, an additional procedure that should be anticipated in lifetime counseling (42).

## 11 Special clinical scenarios

In high and super-high myopia, retinal, macular, and optic nerve disease may limit visual potential independently of refraction. ICL corrects ametropia, not pathological myopia (31). Concave iris or atypical posterior chamber morphology requires careful imaging because predicted support and vault may be unreliable; unfavorable anatomy may justify deferring surgery (43).

Presbyopia-age candidates require discussion of reading needs, tolerance of monovision, and future cataract surgery. Small comparative studies of ICL and FS-LASIK monovision provide preliminary evidence, not a preferred strategy for all older patients (11, 12). Clear-lens status and anatomical eligibility remain necessary even within an approved age range.

## 12 Selection against corneal and lens alternatives

Thin corneas, high refractive correction, or ocular surface vulnerability may favor ICL, while shallow chambers, inadequate ECD, narrow angles, and lens opacity favor another pathway. FS-LASIK, SMILE, and TransPRK avoid intraocular implant risks but have different tissue, flap, surface-healing, and dry-eye trade-offs (5, 32, 33). Refractive lens exchange removes accommodation and needs particular caution in young high myopes. Patient priorities and suitability for each procedure should determine selection; tissue preservation alone cannot override pIOL safety boundaries.

## 13 Artificial intelligence in individualized planning

AI can combine refraction, lens parameters, biometry, and anterior segment imaging to predict postoperative refraction or vault and flag eyes at risk of an extreme vault. In 1,745 eyes, AS-OCT-based machine learning improved vault prediction over the conventional nomogram (44). Another 707-eye study reported a test-set XGBoost mean absolute vault error of 121.70 micrometers, but 95% agreement limits remained approximately -307 to +257 micrometers (45). Clinically important individual errors can therefore remain despite favorable average performance.

Potential applications include candidate size/power comparison, image-derived anatomical features, and risk alerts integrated into surgical planning (26, 28, 46). Current evidence is largely retrospective, with limited representation of hyperopic eyes, unusual anatomy, and underrepresented populations. Patient-level data splitting is necessary to avoid leakage between fellow eyes. Models require multicenter external validation, device-specific calibration, standardized timing and vault measurement, uncertainty reporting, and prospective evidence that their use reduces adverse outcomes. Human review remains essential when predictions conflict with imaging or manufacturer eligibility.

## 14 Discussion and future perspectives

Individualized sizing and power selection, refined haptic/optic designs, and AI-assisted planning should be evaluated against prospective refractive and safety endpoints, rather than predicted vault alone. Larger or presbyopia-oriented optics may improve particular visual needs, but their benefits, dysphotopsia, and anatomical effects require model-specific evaluation (2, 3, 16). Newer acrylic platforms and hyperopic designs need longer follow-up and adequately powered comparisons before safety equivalence can be assumed.

Registries should link baseline anatomy to vault trajectories, endothelial loss, cataract, IOP events, toric rotation, patient-reported symptoms, and explantation. Long-term endothelial reserve and subsequent cataract surgery are particularly important in young recipients (8, 36, 42). This narrative synthesis has selection and publication-bias limitations, does not formally grade evidence, and cannot validate the proposed framework. Numerical boundaries aid consistent reporting but must be interpreted with device labeling and individual anatomy.

## 15 Conclusion

ICL provides effective additive correction for appropriately selected phakic patients, especially those with high myopia and limited corneal options. Measurable eligibility, separate power and size planning, context-sensitive vault interpretation, and long-term monitoring define its safety boundaries. Hyperopic lenses and alternative pIOLs broaden the available options but have distinct anatomy, designs, and evidence gaps. AI may assist individualized planning, while external validation and prospective safety assessment remain necessary.
