# Supplementary material for: Implantable collamer lens surgery for high myopia: safety boundaries, vault management, and patient selection
Source: Front Ophthalmol (Lausanne). 2026 Oct 27;6:1908210. doi: 10.3389/fopht.2026.1908210 (PMC13749031; doi:10.3389/fopht.2026.1908210)
Supplement: Supplementary file 1 [file Table1.docx]

Supplementary Comparisons for ICL Patient Selection and Planning

Implantable Collamer Lens Surgery for High Myopia: Safety Boundaries, Vault Management, and Patient Selection

Manuscript 1908210 | Reference numbers correspond to the revised manuscript.

# Table S1 Phakic IOL Platforms

| **Platform** | **Design and optics** | **Indications and anatomical support** | **Clinical evidence and safety limits** |
| --- | --- | --- | --- |
| EVO/EVO+ ICL and TICL | Collamer plate-haptic lens; 0.36-mm central port. US labeling optic diameter: EVO 4.9-5.8 mm, EVO+ 5.0-6.1 mm, power-dependent. Toric variants correct cylinder (3,9). | Myopia/myopic astigmatism under local labeling. Posterior chamber support; compression and haptic position shape vault. | Extensive cohort and review evidence. Good selected-eye outcomes; cataract, ECD loss, IOP events, and dysphotopsia remain possible (7,8). |
| Non-port ICL including hyperopic designs | Collamer plate-haptic optic without central port; published hyperopic designs include spherical V3 and toric V4B (13,14). | Myopic or positive-power lenses according to model/market. Posterior chamber support; peripheral iridotomy required. | Hyperopic evidence is smaller. A 20-eye toric series showed refractive improvement and mean 1-year vault of 613 micrometers. Older-model long-term results cannot establish EVO-equivalent safety (13,14). |
| IPCL | Reinforced hydrophilic acrylic with distinct haptic geometry. V2.0 myopic central-port design; 6.2-mm optic in the comparative study. Toric and diffractive variants are reported (16,17). | Refractive range and hyperopic/diffractive availability depend on model/market. Sulcus support; use platform-specific sizing. | 60-eye randomized ICL/IPCL comparison: visual improvement in both groups at 12 months, without detected adverse-effect difference. Insufficient power for rare/late risk equivalence (17). |
| Eyecryl | Hydrophilic acrylic posterior chamber plate-haptic lens; central-port configuration depends on model (18,19). | Mainly myopia and myopic astigmatism; model-dependent toric availability. Sulcus support and distinct haptic mechanics. | A comparative study found different vault symmetry/light responses from ICL; clinical implications require confirmation (19). |
| Artisan and Artiflex | Artisan: rigid PMMA optic. Artiflex: foldable silicone optic with PMMA haptics (18). | Anterior chamber iris enclavation. Refractive indications differ by model; endothelial clearance is central. | No posterior chamber vault sizing. Consider endothelial loss, iris/fixation complications, and displacement; follow platform-specific criteria (18). |

S1. Published designs and clinical evidence are compared, rather than assuming that market availability or approval is uniform. Optical range, optic diameter, central-port status, and iridotomy requirements must be checked for the selected model. Short comparisons without a detected difference cannot establish rare-event or long-term equivalence.

# Table S2 Power Calculation and Vault Prediction

| **Method and endpoint** | **Main inputs** | **Reported performance** | **Clinical interpretation** |
| --- | --- | --- | --- |
| Manufacturer modified vergence Refraction/power | Refraction, vertex distance, keratometry, ACD, target; cylinder/axis for toric planning (1,26). | Established comparator. Jiang et al. found overall accuracy comparable to their ML models (26). | A power method, not a vault formula. Regulatory trial results depend on the calculation method used (9). |
| Stacked ML Refraction/power | Age, sphere/cylinder/axis, AL, K, ACD, WTW, pupil/angle measures, and implanted lens sphere/cylinder/axis (26). | 2,767 eyes; best SE MAE 0.339 D (non-toric RF) and 0.325 D (toric XGBoost) (26). | Potential advantage primarily in low-to-moderate myopia. No demonstrated superiority in extreme myopia/hyperopia. |
| NK formula Vault/size | ACW, CLR, and proposed lens size from AS-OCT (22). | 106-eye cohort, 1 month: achieved-minus-predicted bias -104.82 micrometers; 95% limits -600.38 to +391.19 (22). | Signed bias is not MAE. Performance depends on anatomical and lens-size subgroups. |
| KS formula Vault/size | ATA and proposed lens size from AS-OCT (22). | Same 106-eye cohort: bias -68.55 micrometers; 95% limits -628.91 to +491.82 (22). | No significant overall absolute-error difference from NK; neither formula universally superior. |
| AS-OCT ML Vault/size | Preoperative AS-OCT metrics plus clinical/lens parameters (44,46). | 1,745-eye study: ML methods improved prediction over conventional nomogram; RF performed best among tested models (44). | Study-specific ranking; prospective safety benefit is not established. |
| Multibiometric XGBoost Vault | Lens size/power/model, ACD, ATA, CLR, ACW, AL, lens thickness, pupil/angle and other biometric features (45). | 707 eyes; 527-eye test set at 1 week: MAE 121.70 micrometers; RMSE 148.87 micrometers (45). | 95% limits approximately -307 to +257 micrometers. Single-center test performance is not external validation. |

S2. SE: spherical equivalent; MAE: mean absolute error; RF: random forest; RMSE: root mean square error. AL: axial length; ACW: anterior chamber width; ATA: angle-to-angle distance; CLR: crystalline lens rise; WTW: white-to-white distance. Refractive errors are in diopters; vault errors are in micrometers. Signed bias, MAE, RMSE, and agreement limits are different statistics. Results from separate cohorts and time points should not be ranked as a head-to-head comparison. The NK/KS entries are a direct comparison within one study.
